# Exploring beam size measurement based on the Talbot effect at BEPCII

**DOI:** 10.1107/S1600577523006355

**Published:** 2023-08-23

**Authors:** Wan Zhang, Dechong Zhu, Yanfeng Sui, Junhui Yue, Jianshe Cao, Jun He

**Affiliations:** a Institute of High Energy Physics, Chinese Academy of Sciences, Beijing 100049, People’s Republic of China; b University of Chinese Academy of Sciences, Beijing 100049, People’s Republic of China; Tohoku University, Japan

**Keywords:** Talbot effect, interferograms visibility, coherence length, beam size measurement, synchrotron radiation light source

## Abstract

Vertical beam size measurements were carried out at Beijing Electron–Positron Collider II (BEPCII) using a phase grating and an absorption grating based on the Talbot effect. The transverse coherence of synchrotron radiation is closely related to beam size. Due to the partial coherence of the synchrotron radiation source, the coherence length can be calculated by measuring the visibility decay of interferograms recorded at different distances behind the gratings. A vertical beam size of 68.19 ± 2 µm was obtained based on the relationship between the coherence length and beam size at the 3W1 beamline of BEPCII. A comparison of the vertical emittance derived from the grating Talbot method and the synchrotron radiation visible light interferometer method was presented. The vertical emittances from the two methods are 1.41 nm rad and 1.40 nm rad, respectively. The 0.1% difference indicates that the grating Talbot method for beam size measurement is reliable. This technique has great potential for small beam size measurement of fourth-generation synchrotron radiation light sources, considering its small diffraction limitation and simple experimental setups.

## Abstract 2

A method based on the Talbot effect for accurate beam size measurement has been implemented at Beijing Electron–Positron Collider II. This technique has great prospects for application in small beam size measurement of fourth-generation synchrotron light sources, benefiting from its small diffraction limitation.

## 1. Introduction

The requirements of synchrotron radiation facilities for high coherence, high brightness and small beam sizes have been proposed in some X-ray beamlines for applications such as X-ray phase contrast imaging, coherent X-ray diffraction imaging and X-ray holography (Pogoreliy *et al.*, 2009 ▸; Yang *et al.*, 2013 ▸; Sakdinawat & Attwood, 2010 ▸). Beam size measurement plays a critical role in beam diagnostics and beam dynamics studies (Samadi *et al.*, 2020 ▸; Koopmans *et al.*, 2019 ▸). Accurate measurements of small beam size are becoming increasingly necessary.

Radiation-based techniques for measuring electron beam sizes are widely used as beam diagnostics at existing synchrotron sources (Garg *et al.*, 2018 ▸; Mitsuhashi, 1998 ▸). Thanks to their short wavelength, X-rays can greatly improve the imaging resolution to meet small beam size measurements. X-ray pinhole imaging, as a common method, has been applied at Diamond Light Source, European Synchrotron Radiation Facility and so on (Thomas *et al.*, 2010 ▸; Garg *et al.*, 2014 ▸; Leitenberger *et al.*, 2003 ▸). It is characterized by real-time and high measurement accuracy. However, it does not work for extremely small beam sizes due to the diffraction limitation. X-ray Fresnel zone plate imaging and Kirkpatrick–Baez mirror focusing imaging show advantages in micrometre-scale beam size measurement. However, they suffer from special beamline design and high machining accuracy (Suzuki *et al.*, 2001 ▸; Alatas *et al.*, 2011 ▸). In recent years, some new measurement systems have been applied to beam size measurement. A method for simultaneously measuring the vertical position and vertical divergence angle was proposed by means of X-ray diffraction and materials absorption edges at Canadian Light Source (Samadi *et al.*, 2019 ▸). However, a smaller beam size is difficult to measure due to diffraction effects. The following year, an X-ray tandem double-slit optical system was proposed to measure beam size and divergence angle at SPring-8 (Kagoshima *et al.*, 2020 ▸). Although this method can measure beam size in micrometres, the tandem double-slit needs to be aligned, which is difficult.

For the case of synchrotron radiation sources with high coherence, it is feasible to derive the beam size by spatial coherence. One of the Talbot effect applications is focused on spatial coherence measurements of X-rays in synchrotron radiation sources. The spatial coherence of X-rays emitted from a bending magnet has been measured using the Talbot effect of a π/2 phase checkerboard grating and a π/2 phase circular grating at Advanced Photon Source (Marathe *et al.*, 2014 ▸; Shi *et al.*, 2014 ▸). In these papers, the Talbot effect is mainly used to study the coherence of X-rays. Given the relationship between spatial coherence and source size, the grating Talbot method can also be used to measure beam size owing to its small diffraction limitation. Most importantly, the experimental setup is simple, needing only gratings, a displacement platform and an X-ray camera without special design in the beamline front-end.

At BEPCII, we measured the vertical beam size at 3W1 beamline by employing the grating Talbot effect. Partially coherent quasi-monochromatic X-rays have been employed in this experiment. The vertical beam size is calculated successfully from the self-image interference fringes of a grating interferometer. Then the vertical emittance of the storage ring is calculated by the vertical beam size and β function. As a contrast, the vertical emittance from a bending magnet is derived using the visible-light interference method. An extremely small difference between the two vertical emittances is presented, which illustrates that the grating Talbot effect method has great potential for measuring beam size.

## 2. Measurement system

### 2.1. Theory background

In 1836, Talbot found that a monochromatic parallel beam transmitting through a grating vertically will generate a series of grating images at certain distances behind the grating, which is called the Talbot effect (Talbot, 1836 ▸; Born & Wolf, 1999 ▸). There is a specific relationship between the visibility of the Talbot image and the complex coherence function of the light source (Cloetens *et al.*, 1997 ▸; Guigay *et al.*, 2004 ▸).

The interference intensity of two beams from an extended source at any point can be written as

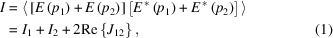

where *E*(*p*_1_) and *E*(*p*_2_) are electric fields formed by two points on an extended source, and *J*_12_ is the mutual intensity function of the two light beams from the two points (Pfeiffer *et al.*, 2005 ▸).

The normalized mutual intensity function *j*_12_, also called the complex coherence function, is expressed as

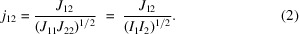

Combining equations (1) and (2),

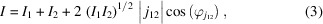

where 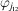 = Arg(*j*_12_). The third term of equation (3) expresses the interference effect. |*j*_12_| takes the value 1 corresponding to the complete coherence of the two beams and takes the value 0 corresponding to the complete incoherence. In the case of partial coherence, it takes a value between 0 and 1.

The complex coherence function can be calculated from the visibility measurement of interference fringes formed by two light beams. The visibility can be expressed as

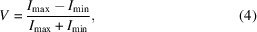

where *I*_max_ and *I*_min_ are the maxima and minima intensity of interference fringes, respectively. They can be found from equation (3),

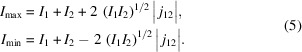

Inserting equation (5) into equation (4),

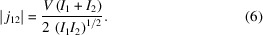

For a phase grating, *I*_1_ = *I*_2_, and equation (6) can be rewritten as

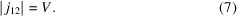

That is to say, the visibility of fringes is equal to the coherence degree of the beam.

The intensity of synchrotron radiation shows a good approximation to a Gaussian distribution, and can be written as

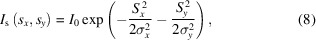

where *s*_*x*_, *s*_*y*_ are the coordinates in the source plane, and σ_*x*_ and σ_*y*_ are the source size along the horizontal and vertical directions, respectively.

The complex coherence function is also a Gaussian distribution according to the propagation theory of the mutual intensity function, which is expressed as

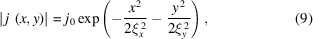




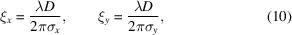

where *x* and *y* are the horizontal and vertical axes, respectively, which are perpendicular to the direction of beam propagation, ξ_*x*_ and ξ_*y*_ are the coherence length in the *x* and *y* directions, respectively, *D* is the beam propagation distance from the source, and λ is the transmission wavelength.

In this experiment, the measured visibilities are from a series of grating self-imaging interference fringes at different *d*, the distance between grating and camera. For a π/2 phase-shift grating, the interference fringes are formed by the neighboring diffraction orders, thus the coordinates in equation (9) can be rewritten in terms of *d* (Zanette *et al.*, 2010 ▸),

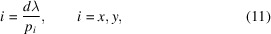

where *p*_*i*_ is the period of the interference fringes along the *i* direction. As a consequence, equation (9) can be expressed as a function of *d*,

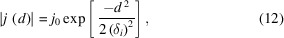

where δ_*i*_ is the width of the Gaussian envelop function along the *i* direction. Combining equations (9) and (11), the coherence length can be expressed as

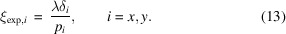

By inserting the measured coherence length ξ_exp,*i*_ into equation (10), the source size can be calculated.

### 2.2. Energy bandwidth of X-rays

**Figure 1:**
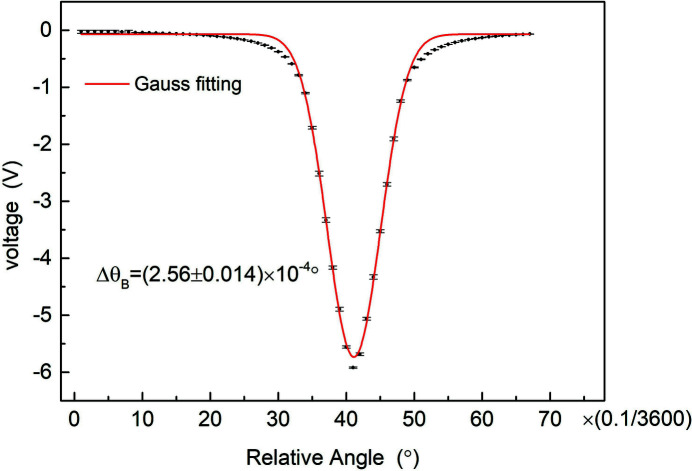
Ionizing voltage of the ion chamber as a function of the relative angle between two crystals.

The experiment was performed at the 3W1 beamline of BEPCII. X-rays of 15 keV were obtained after passing through a Si(311) double-crystal monochromator (DCM). The X-ray energy bandwidth has an effect on the beam size measurement which has to be considered. It can be measured by scanning the second crystal angle of the DCM (Liermann *et al.*, 2015 ▸). An ion chamber as a detector provides a current which is proportional to the absolute incident photon flux. The incident photon flux is changed with the relative angle between the two crystals. Then the current can be converted to a voltage which is a function of the relative angle between the two crystals. The corresponding voltage curve is illustrated in Fig. 1 ▸. The horizontal coordinate is the relative angle between the two crystals, and the vertical coordinate is the ionizing voltage generated by the X-rays. The full width at half-maximum (FWHM) of the Gaussian distribution is Δθ_B_ = (2.56 ± 0.014) × 10^−4^ degrees. According to the Bragg diffraction formula 2*d*sinθ_B_ = *m*λ [where *m* is an integer, *d* = 1/(*h* ^2^ + *k* ^2^ + *l* ^2^)^1/2^, for Si(311), *h* = 3, *k* = 1, *l* = 1] and the photon energy formula *E* = *hc*/λ, an energy bandwidth Δ*E*/*E* of (1.7 ± 0.001) × 10^−5^ is calculated according to the equation Δ*E*/*E* = Δθ_B_/tanθ_B_ (Yang *et al.*, 2020 ▸).

### 2.3. Experimental setups

**Figure 2:**
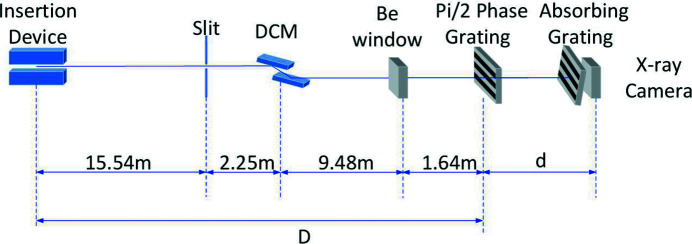
Schematic of the experimental setup.

Figure 2 ▸ shows a schematic of the experimental setup. A 9 mm × 1.3 mm slit is placed 15.54 m from the source. The DCM is located downstream and 2.25 m from the slit. A beryllium window is 9.48 m away from the DCM. The 1-D π/2 phase grating is placed 28.91 m from the source. The distance between the 1-D absorption grating and camera is about 0.01 m. The X-ray camera consists of a CCD, a lens and a LYSO (lutetium-yttrium oxyorthosilicate) scintillator which is used to convert X-rays into visible light. The CCD pixel size is 6.5 µm × 6.5 µm and the lens magnification is 10. The resolution of the X-ray camera is 0.65 µm.

**Figure 3:**
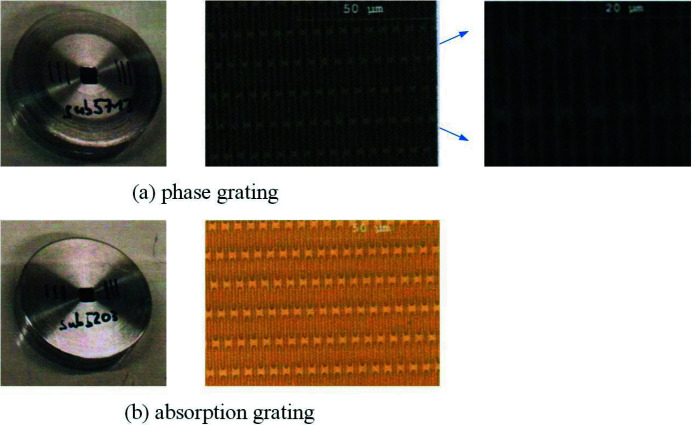
Photographs and scanning electron microscope images of (*a*) the phase grating and (*b*) the absorption grating.

The grating interferometer, consisting of a phase grating and an absorption grating, is the key part of the experiment. Figure 3 ▸ shows photographs and scanning electron microscope images of two gratings fabricated by Microworks, Germany. The black lines in the photographs indicate the direction of the grating lines. In the photographs, there are some horizontal structures. These horizontal structures are the nodes generated during the grating-making process. It can be seen in the zoomed image (Fig. 3 ▸, top right) that the grating lines are still vertical.

Some fabricating parameters of the gratings are shown in Table 1 ▸. The gratings’ period is 2.4 µm and the duty cycle is 0.5 for uniform self-imaging fringes. The grating lines of the phase grating and absorption grating are made of polymer and gold, respectively. The substrates for both gratings are made of polyimide. The height of the polymer of the phase grating is 18.6 µm and the height of the gold of the absorption grating is 14 µm, which is determined by the phase shift and X-ray energy of 15 keV.

**Figure 4:**
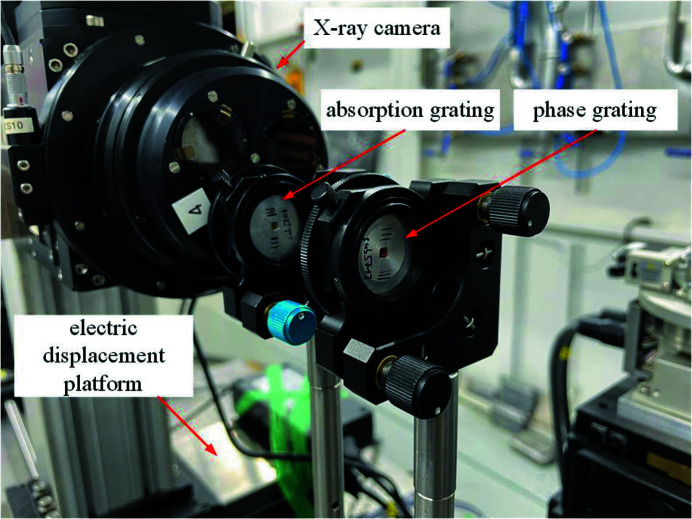
The experimental grating interferometer.

The grating interferometer device is shown in Fig. 4 ▸. The phase grating is fixed on an optical platform. The absorption grating and X-ray camera move together along the direction of light propagation. The phase grating produces self-images at fractional Talbot distances (*d*_*n*_) following the equation *d*_*n*_ = *n*(*p* ^2^/2λ), where *n* = 0.5, 1.5, 2.5… (Pfeiffer *et al.*, 2005 ▸). Each self-image is superimposed with the absorption grating forming Moire fringes which are detected by an X-ray camera. The grating lines of the phase grating are in the horizontal direction, and there is a relatively small angle between the grating lines of the two gratings.

The interference fringes represent the coherence of the source in the vertical direction, and the vertical beam size can be calculated from the coherence. Measurements are performed by acquiring interferograms at multiple detector positions from close to the grating to the maximum distance.

## 3. Results and analysis

### 3.1. Grating self-imaging fringes

**Figure 5:**
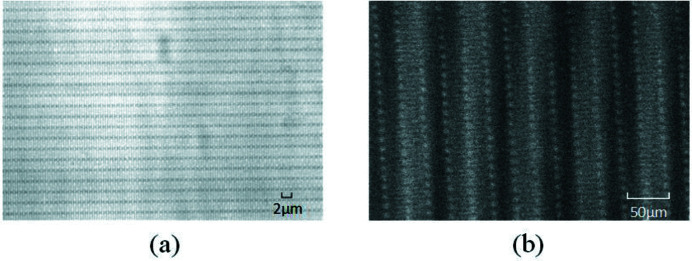
Self-image of the phase grating (*a*) and the Moire fringes formed by the phase grating and the absorbing grating (*b*).

A self-image of the phase grating is shown in Fig. 5 ▸(*a*). It is similar to the photograph of the scanning electron microscope of the phase grating. However, the contrast between light and dark stripes is very small. In order to achieve obvious interference fringes, an absorption grating is introduced. Figure 5 ▸(*b*) shows the Moire fringes formed by the phase grating and the absorbing grating. The period can be calculated by the Moire fringe period formula, *B* = *p*/sinθ, where *p* is the grating period and θ is the relative angle of the grating lines of the two gratings. Notably, the interference image has a more significant period benefitting from extraction of the visibility.

**Figure 6:**
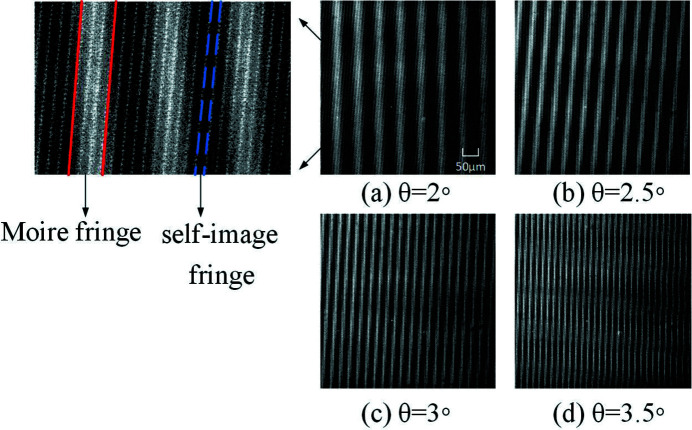
Moire fringes with (*a*) θ = 2°, (*b*) θ = 2.5°, (*c*) θ = 3° and (*d*) θ = 3.5°.

A Moire fringe interference image for different angles θ is shown in Fig. 6 ▸. A larger angle θ corresponds to a smaller fringe period. According to interference theory, the visibility of the self-imaging fringe generated by a single phase grating is the same as the visibility of the Moire fringes. Thus, in the following experiments, the two-grating structure with θ = 2° is selected due to its larger fringe period. In the top-left image of Fig. 6 ▸, the fringe highlighted by the red solid lines is the Moire fringe, which has a large period; the fringe highlighted by the blue dotted lines is the self-image of the grating, which has a small period.

### 3.2. Visibility and vertical beam size

**Figure 7:**
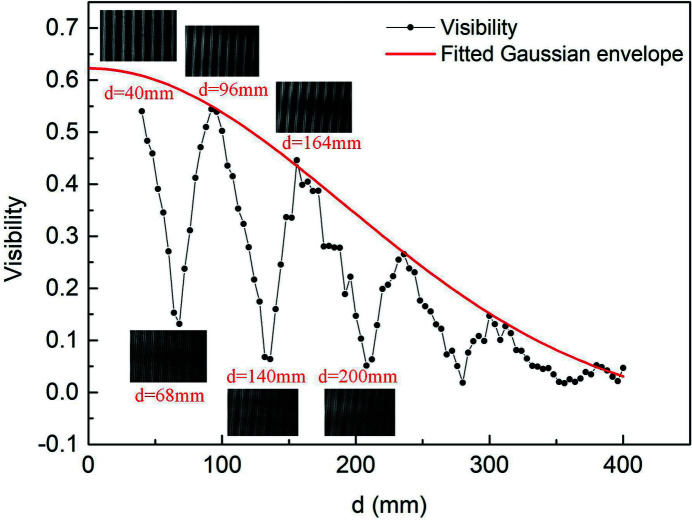
Visibility as a function of detection distance *d*.

The visibility as a function of detection distance *d* is shown in Fig. 7 ▸. The sinusoidal oscillation of the visibility is due to the fractional Talbot effect imparted by the phase grating, and it decreases gradually because of the partial coherence of the source. Some images of the interferograms at different distances *d* are displayed. The images have higher visibilities at *d* = 40 mm, 96 mm and 164 mm near the fractional Talbot distances. On the contrary, the images at *d* = 68 mm, 140 mm and 200 mm have lower visibilities due to the weak self-images effect. The two kinds of images appear alternately with increasing *d*, which is consistent with theoretical expectations.

**Figure 8:**
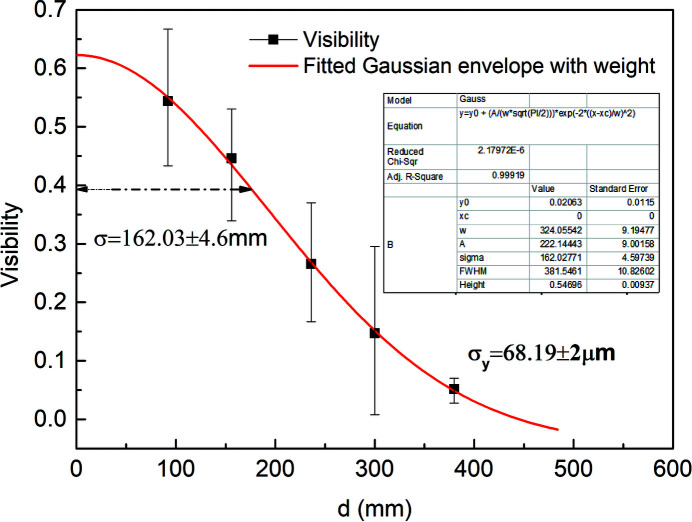
Fitted Gaussian envelope curve of the maximum visibility points.

The transverse coherence of the X-ray wavefront is related to the width of the envelope function, which can be modeled as Gaussian according to equation (10). Figure 8 ▸ shows the fitted Gaussian envelope function drawn through the maximum visibility points. The first fractional Talbot distance about 34 mm is eliminated because it is smaller than the minimum distance between two gratings. The *R* ^2^ value of the fitted Gaussian function is greater than 0.99, presenting a high fitting accuracy. The transverse coherence of the X-rays can be calculated by utilizing the width of the envelope function according to the theory in Section 2. The width of the envelope function σ is 162.03 ± 4.6 mm, and a vertical coherence length of 5.58 ± 0.16 µm on the phase grating plane is achieved. The vertical beam size is σ_*y*_ = 68.19 ± 2 µm by inserting the vertical coherence length into equation (10). In the experiment, the visibility is averaged over multiple measurements, and error bars are introduced. The vertical beam size error is calculated considering the visibility error and the error caused by the energy bandwidth. An error of ±2 µm indicates that this method has a small measurement error. The accuracy of this method is evaluated by comparing the vertical emittances measured by the two methods discussed in the following section.

### 3.3. Comparing methods – double-slit interference

The experimental results are assessed by comparing two vertical emittances from two methods – the grating self-imaging method (at beamline 3W1 with wiggler source) and the synchrotron radiation (SR) interferometer method (at a visible-light diagnostics beamline with bending magnet source). The vertical emittance ɛ_*y*_ is a constant for an accelerator. It can be calculated by vertical beam size and Lattice parameter β_*y*_ at the source point according to the formula 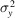 = ɛ_*y*_β_*y*_.

**Figure 9:**
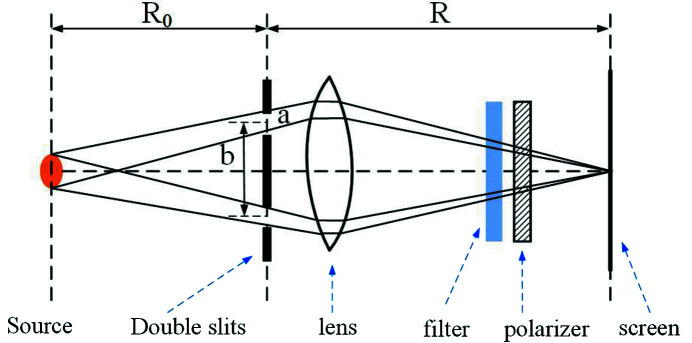
Diagram of double-slit interference.

A double-slit interferometer is applied to measure the beam size at the visible-light diagnostics beamline. The principle of double-slit interference is based on Van Citterut–Zernike theory. The spatial coherence of a finite-size light source is the Fourier transform of the light source size, expressed as (Wang *et al.*, 2013 ▸)

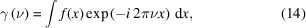

where γ(ν) is the spatial coherence, *f*(*x*) is the normalized source distribution and ν is the spatial frequency. A diagram of double-slit interference is shown in Fig. 9 ▸. The intensity of the interference pattern measured in the detector plane is

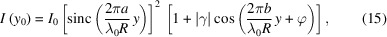

where *a* is half of the single slit height, *b* is the distance between the centers of two slits, *R* is the distance from the double-slit to the detector, λ_0_ is the wavelength of the observation, ϕ denotes the fringe phase and *I*_0_ is the sum of incoherent intensities from the two slits. The spatial frequency ν is expressed as ν = *b*/λ_0_ *R*_0_, where *R*_0_ is the distance from the source to the double slit.

The spatial coherence γ can be calculated by fitting the intensity distribution of the interference fringes according to equation (15). The cross-sectional distribution of the original source is obtained from the inverse Fourier transform of the different spatial coherences measured by the corresponding double-slit spacing *b* (meaning different spatial frequency). The cross-sectional distribution of the beam in the storage ring approximates a Gaussian distribution. To simplify the complex calculation process, a Gaussian function is substituted into the coherence calculation equation (14), and the relationship between beam size σ and spatial coherence γ can be expressed as

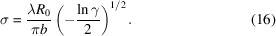

In general, the beam size is calculated with the spatial coherence measured by a fixed slit spacing according to equation (16).

At the visible-light diagnostics beamline, we measured the vertical beam size with a double-slit interferometer with a wavelength of 550 nm. Taking into account the relationship between light intensity, single-slit width and double-slit distance, a slit width of 0.8 mm and a double-slit distance of 4 mm are selected. The distance from the source to the double-slit, *R*_0_, is 5.85 m.

**Figure 10:**
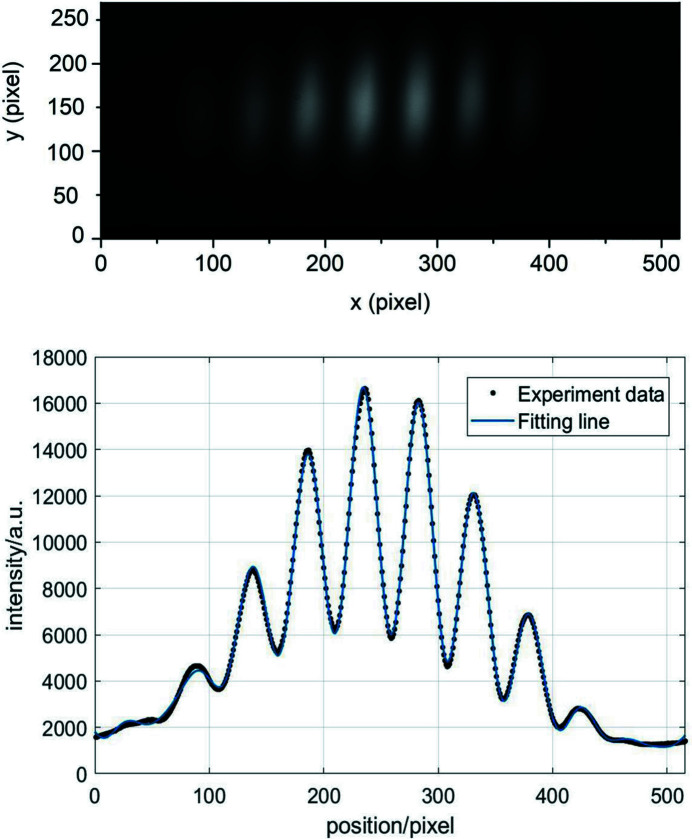
Interference fringes and intensity distribution curve for vertical beam size measurement.

**Figure 11:**
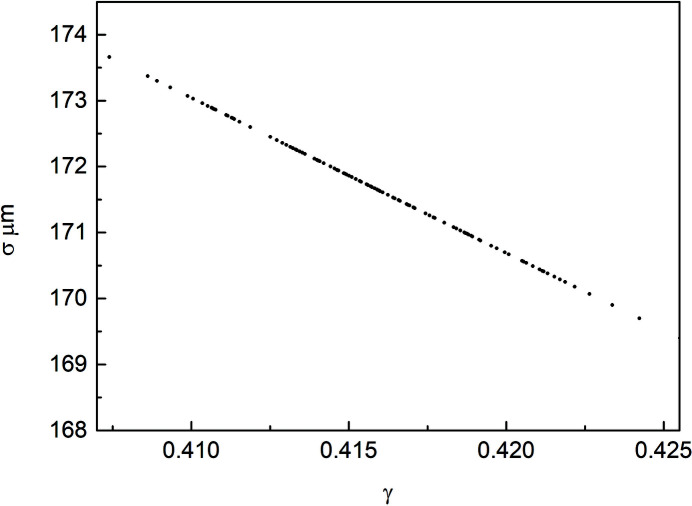
Vertical beam size as a function of γ for a period of time.

Figure 10 ▸ shows the interference fringes collected in the vertical beam size measurement experiment. The raw data are a 2D matrix with 250 rows and 520 columns. With integration along the *y*-direction, the intensity distribution curve along the *x*-direction is shown in Fig. 10 ▸. By fitting the curve using equation (15) (where the fitting line is expressed in Fig. 10 ▸), γ is found to be 0.41. A vertical beam size of 172 µm is derived by substituting γ into equation (16). The vertical beam size as a function of γ for a period of time is show in Fig. 11 ▸.

Finally, a comparison of two vertical emittances from the two methods is presented in Table 2 ▸. The measured vertical emittance is 1.41 nm rad at beamline 3W1 and 1.40 nm rad at the visible-light diagnostics beamline. The extremely small difference, about 0.1%, indicates that the grating self-imaging method can be used to measure beam size at a synchrotron radiation source. Possible future measurements for smaller beam sizes of fourth-generation synchrotron light sources with higher light source coherence are being considered due to the small diffraction limitation and simple experimental setups of the proposed method.

## 4. Conclusion

A method based on the grating self-imaging effect for accurate beam size measurement has been implemented at BEPCII. Due to the partial coherence of X-rays, the transverse coherence of the wavefront has a relationship with the visibility of self-imaging interferograms. The beam size can be derived from the visibility.

In this paper, the vertical beam size from a wiggler source is measured by extracting visibilities of interferograms formed by a phase grating and absorption grating. The interferograms, also called Moiré fringes, have a larger fringe period which is conducive to visibility extraction. The transverse coherence of 5.58 ± 0.16 µm in the vertical direction in the plane of the phase grating is obtained from the width of the fitted Gaussian envelope function. Then the vertical beam size is calculated to be 68.19 ± 2 µm. Finally, to evaluate the accuracy of the experimental results, the vertical emittances derived from the grating self-imaging method and SR interferometer method are compared, which are 1.41 nm rad and 1.40 nm rad, respectively. Thanks to the extremely small difference, about 0.1%, the grating self-imaging method for measuring beam size is considered to be reliable and accurate. This technique has great prospects for application in small beam size measurement of fourth-generation synchrotron light sources, benefiting from the small influence of diffraction limitation.

## Figures and Tables

**Table 1:** Some fabricating parameters of the gratings

	Phase grating	Absorption grating
Period	2.4 µm	2.4 µm
Duty cycle	0.53 ± 0.01	0.51 ± 0.01
Area	>2.5 mm × 2.5 mm	>2.5 mm × 2.5 mm
Height	Polymer, 18.6 µm	Gold, 14 ± 1 µm
Substrate	10 µm polyimide	10 µm polyimide

**Table 2:** Comparison of vertical emittance derived from the two methods

Method	Grating self-imaging	SR interferometer
Beamline	3W1 beamline	Visible-light diagnostics beamline
β_*y*_	3.29 m	20.98 m
σ_*y*_	68.19 µm	171.56 µm
ɛ_*y*_	1.41 nm rad	1.40 nm rad
